# Severe Anemia and Acute Kidney Injury After Pegloticase in a Patient With Glucose-6-Phosphate Dehydrogenase Deficiency

**DOI:** 10.7759/cureus.105601

**Published:** 2026-03-21

**Authors:** Noor Zainab, Tamer Zahdeh, Khaled W Erekat, Michelle N Ritota, Mahmoud El Hajj, Aiswarya Nair, Umair Siddiqi

**Affiliations:** 1 Internal Medicine, Montefiore St. Luke's Cornwall, Newburgh, USA; 2 General Surgery, King Hussein Medical Center, Amman, JOR; 3 Internal Medicine, St. George's University, True Blue, GRD; 4 Internal Medicine, UT Health San Antonio, San Antonio, USA; 5 Infectious Diseases, University of Connecticut, Storrs, USA

**Keywords:** acute hemolytic anemia, acute kidney injury, g6pd deficiency, krystexxa (pegloticase), normocytic normochromic anemia

## Abstract

Pegloticase is an effective therapy for refractory gout but carries a risk of oxidative hemolysis in patients with glucose-6-phosphate dehydrogenase (G6PD) deficiency. Reported cases of hemolytic complications remain limited, particularly those with delayed presentation, where traditional markers such as haptoglobin and bilirubin may have normalized, making diagnosis challenging. We describe a 61-year-old female with known G6PD deficiency who presented with near syncope, nausea, vomiting, and volume depletion four days after receiving her first dose of intravenous pegloticase and recently starting mycophenolate. Initial evaluation revealed severe anemia (hemoglobin 5.1 g/dL) and acute kidney injury (creatinine 9.23 mg/dL). Hemolysis markers were not overtly diagnostic, with normal haptoglobin and bilirubin; however, urinalysis revealed heme-positive urine with no red blood cells, a finding highly suggestive of hemoglobinuria from recent intravascular hemolysis rather than true hematuria. An extensive workup for anemia and acute kidney injury was otherwise unrevealing. Given the temporal association with pegloticase exposure and underlying G6PD deficiency, oxidative hemolysis with concomitant prerenal acute kidney injury was suspected. The patient improved with discontinuation of the offending agents, blood transfusion, and supportive care, with stabilization of hemoglobin and gradual recovery of renal function. This case highlights the importance of recognizing delayed or atypical presentations of drug-induced hemolysis, in which traditional markers may be normal at the time of evaluation. Clinicians should maintain a high index of suspicion for hemolysis after pegloticase administration in G6PD-deficient patients and ensure appropriate screening prior to therapy.

## Introduction

Pegloticase is a recombinant uricase enzyme approved for the treatment of refractory chronic gout, particularly in patients who fail conventional urate-lowering therapies. By catalyzing the oxidation of uric acid to allantoin, pegloticase enables rapid urate reduction but also generates hydrogen peroxide as a metabolic byproduct. In patients with glucose-6-phosphate dehydrogenase (G6PD) deficiency, impaired NADPH production via the pentose phosphate pathway leads to inadequate neutralization of hydrogen peroxide, resulting in oxidative damage to erythrocyte membranes and hemoglobin, ultimately causing acute intravascular hemolysis. This oxidative burden places patients with G6PD deficiency at increased risk of hemolytic anemia and methemoglobinemia, and current prescribing guidelines recommend routine G6PD screening prior to initiation of therapy [1-3].

Although the risk of hemolysis with uricase-based therapies is well established from experience with rasburicase, and several cases of pegloticase-associated hemolysis have been reported, real-world presentations with delayed onset and equivocal laboratory markers remain underrecognized and diagnostically challenging, when classic laboratory markers of hemolysis such as haptoglobin and indirect bilirubin may have normalized due to hepatic clearance and compensatory synthesis [4,5]. In such delayed presentations, clues including hemoglobinuria (heme-positive urine without red blood cells), unexplained acute anemia, and temporal association with known oxidative triggers such as pegloticase may be more informative than traditional hemolysis markers. Similar diagnostic challenges have been reported in other hematologic conditions, where rare but fatal complications may present with nonspecific symptoms and be overlooked if clinical suspicion is low.

We present a case of severe anemia and acute kidney injury (AKI) in a patient with known G6PD deficiency shortly after receiving pegloticase therapy. This case highlights the potential for delayed or atypical presentations of drug-induced oxidative hemolysis and the importance of maintaining clinical suspicion despite equivocal hemolysis markers, with emphasis on the need for appropriate G6PD screening and monitoring prior to pegloticase administration in high-risk populations.

## Case presentation

A 61-year-old female with a past medical history significant for hypertension, rheumatoid arthritis, gout, left renal cell carcinoma status post nephrectomy, uterine fibroids status post hysterectomy, gastroesophageal reflux disease, depression, G6PD deficiency, and morbid obesity (BMI 40 kg/m^2^) was brought to the emergency department by ambulance for a near-syncopal episode preceded by lightheadedness.

She reported a four-day history of persistent nausea, multiple episodes of bilious vomiting, loose bowel movements, and a one-day history of anuria. She denied chest pain, dyspnea, palpitations, vertigo, headache, neck pain, focal weakness, sensory deficits, abdominal or back pain, urinary symptoms, melena, hematuria, recent travel, or sick contacts. Her home medications included valsartan-hydrochlorothiazide, labetalol, methotrexate, allopurinol, escitalopram, and buspirone. Notably, three days prior to symptom onset, she had received her first dose of intravenous pegloticase for refractory gout and was newly started on mycophenolate.

On scene, she was borderline hypotensive with a documented blood pressure of 100/50 mmHg. In the emergency department, her vital signs were as follows: temperature 98.1°F, blood pressure 109/61 mmHg, heart rate 76 beats per minute, respiratory rate 18 breaths per minute, and oxygen saturation 100% on room air. She was alert, awake, and oriented, reporting nausea without other acute complaints. Physical examination was largely unremarkable and without focal abnormalities. She was treated with intravenous ondansetron 4 mg for symptomatic relief and initiated on intravenous isotonic fluid resuscitation.

**Figure 1:**
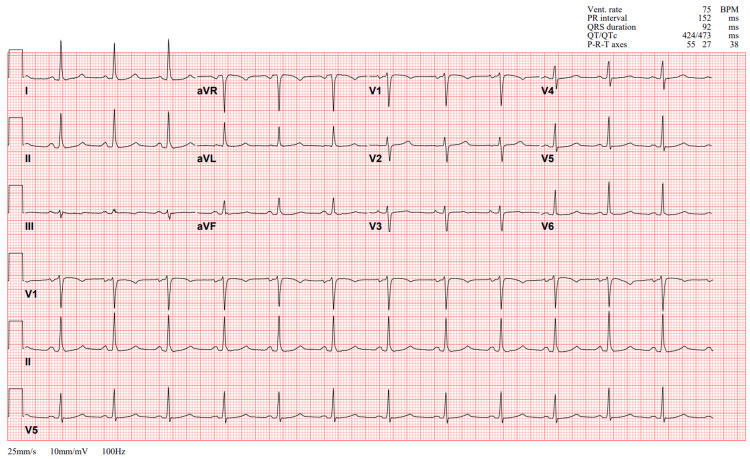
ECG on admission.

**Figure 2:**
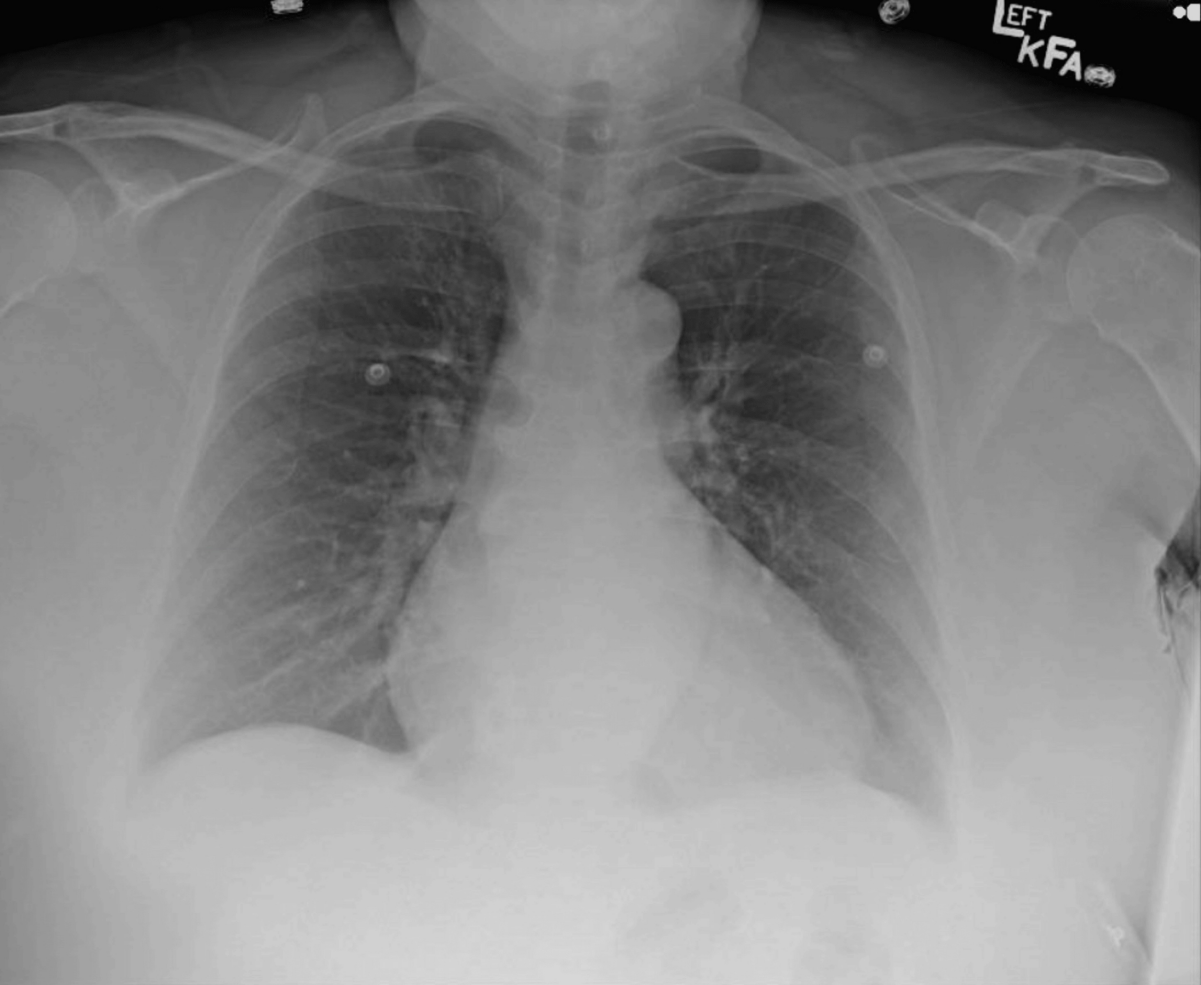
Chest X-ray on admission.

**Figure 3:**
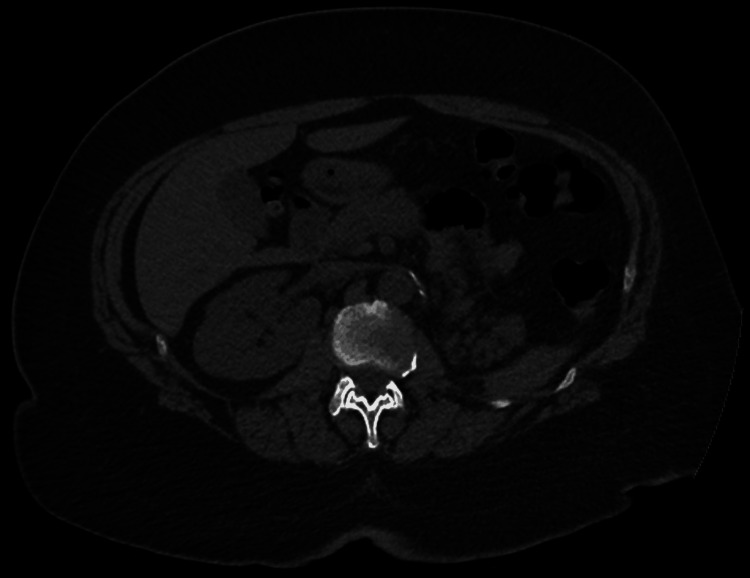
CT abdomen on admission showing a solitary right kidney and a surgically absent left kidney.

Initial blood workup evaluation revealed severe AKI (creatinine of 9.23 mg/dL) and profound anemia (hemoglobin of 5.1 g/dL) (Table 1). Electrocardiography showed a normal sinus rhythm with normal PR, QRS, and QT intervals and no acute ischemic changes (Figure 1). Chest radiography showed no acute cardiopulmonary pathology, including no focal consolidations or pleural effusions (Figure 2). Computed tomography of the abdomen and pelvis without contrast revealed nonspecific bladder wall thickening without evidence of hydroureteronephrosis or bowel wall thickening (Figure 3).

**Table 1:** Initial laboratory and urinalysis findings on presentation. BUN: blood urea nitrogen; eGFR: estimated glomerular filtration rate; CO_2_: bicarbonate; WBC: white blood cell; RBC: red blood cell; MCV: mean corpuscular volume; MCH: mean corpuscular hemoglobin; MCHC: mean corpuscular hemoglobin concentration; RDW: red cell distribution width; AST: aspartate aminotransferase; ALT: alanine aminotransferase; ESR: erythrocyte sedimentation rate; CRP: C-reactive protein; TSH: thyroid-stimulating hormone; HPF: high-power field

Test	Result	Reference Range
Sodium	135 mmol/L	135-145 mmol/L
Potassium	4.2 mmol/L	3.5-5.0 mmol/L
Chloride	105 mmol/L	98-107 mmol/L
CO_2_	21 mmol/L	22-29 mmol/L
Anion Gap	9	8-16
Blood Urea Nitrogen (BUN)	117 mg/dL	7-20 mg/dL
Creatinine	9.23 mg/dL	0.6-1.3 mg/dL
Glucose	98 mg/dL	70-99 mg/dL
Calcium	8.2 mg/dL	8.6-10.2 mg/dL
Magnesium	1.6 mg/dL	1.7-2.2 mg/dL
Phosphorus	5.0 mg/dL	2.5-4.5 mg/dL
eGFR	4.34 mL/min/1.73 m^2^	>60 mL/min/1.73 m^2^
Serum Osmolality	307 mOsm/kg	275-295 mOsm/kg
White Blood Cell Count	7.66 ×10^3^/µL	4.0-11.0 ×10^3^/µL
Red Blood Cell Count	1.86 ×10^6^/µL	4.2-5.9 ×10^6^/µL
Hemoglobin	5.1 g/dL	12-16 g/dL (F)/13.5-17.5 g/dL (M)
Hematocrit	17.0%	36-50%
MCV	91.4 fL	80-100 fL
MCH	27.4 pg	27-33 pg
MCHC	30.0 g/dL	32-36 g/dL
RDW	18.9%	11-15%
Platelet Count	238 ×10^3^/µL	150-450 ×10^3^/µL
Neutrophils	67.9%	40-70%
Lymphocytes	19.3%	20-40%
AST	29 U/L	10-40 U/L
ALT	12 U/L	7-56 U/L
Alkaline Phosphatase	77 U/L	44-147 U/L
Total Bilirubin	0.4 mg/dL	0.1-1.2 mg/dL
Direct Bilirubin	0.08 mg/dL	0.0-0.3 mg/dL
Albumin	3.4 g/dL	3.5-5.0 g/dL
Total Protein	7.4 g/dL	6.0-8.3 g/dL
Globulin	4.0 g/dL	2.0-3.5 g/dL
ESR	41 mm/hr	<20 mm/hr
CRP	0.868 mg/dL	<0.5 mg/dL
Troponin I (High Sensitivity)	18.7 ng/L	<54 ng/L
TSH	0.556 µIU/mL	0.4-4.0 µIU/mL
Vitamin B12	508 pg/mL	200-900 pg/mL
Folate	33.3 ng/mL	2.7-17.0 ng/mL
Urine Appearance	Slightly abnormal	Clear
Urine Color	Yellow	Yellow
Urine Specific Gravity	1.010	1.005-1.030
Urine pH	5.0	4.5-8.0
Urine Glucose	Negative	Negative
Urine Ketones	Negative	Negative
Urine Blood	Moderate	Negative
Urine Leukocyte Esterase	Negative	Negative
Urine Nitrites	Negative	Negative
Urine WBC	6-10/HPF	0-5/HPF
Urine RBC	0-3/HPF	0-3/HPF
Urine Bacteria	Negative	None
Urine Yeast	1	None
Urine Protein	Negative	Negative
Urine Microalbumin	46.5 mg/g	<30 mg/g

The patient received a total of five units of packed red blood cells and was subsequently admitted for further management. Antihypertensive medications were held, and she was started on an intravenous sodium bicarbonate infusion (75 mEq in 5% dextrose at 75 mL/hour). A comprehensive anemia and AKI workup was performed on the following day of admission, as summarized in Table 2.

**Table 2:** Anemia diagnostic workup. LDH: lactate dehydrogenase; TIBC: total iron binding capacity; IFE: immunofixation electrophoresis; CD: cluster of differentiation; G6PD: glucose-6-phosphate dehydrogenase; Hb: hemoglobin

Test	Result	Reference Range
LDH	390 U/L	140-280 U/L
Haptoglobin	73.1 mg/dL	30-200 mg/dL
Direct Coombs Test	Negative	Negative
Reticulocyte %	2.95%	0.5-2.5%
Serum Iron	48 µg/dL	60-170 µg/dL
TIBC	245 µg/dL	250-450 µg/dL
Ferritin	969.7 ng/mL	20-300 ng/mL
Transferrin Saturation	20%	20-50%
Urine Total Protein (24 Hours)	406 mg/day	<150 mg/day
Urine Microalbumin	46.5 mg/g	<30 mg/g
Free Urinary Kappa Light Chains	162.18 mg/L	0-32.9 mg/L (lab dependent)
Free Urinary Lambda Light Chains	65.47 mg/L	0-4.99 mg/L (lab dependent)
Urinary Kappa Excretion/Day	243.27 mg/day	Variable
Urinary Lambda Excretion/Day	98.20 mg/day	Variable
Urine IFE	Negative for monoclonal proteins	Negative
Serum IFE	Normal pattern; no monoclonal proteins	No monoclonal spike
Cold Agglutinins	<1:32	<1:32
Cryoglobulins	Negative (72 hours)	Negative
CD4 Count	528 cells/µL	500-1500 cells/µL
CD8 Count	191 cells/µL	200-900 cells/µL
CD4:CD8 Ratio	2.80	1.0-3.5
Hepatitis B Surface Antigen	Nonreactive	Nonreactive
Hepatitis B Surface Antibody	Nonreactive	Reactive/Nonreactive
Hepatitis C Antibody	Nonreactive	Nonreactive
G6PD	9.8 U/g Hb	9.9-16.6 U/g Hb

Given the temporal association with recent pegloticase administration and initiation of mycophenolate, her symptoms of persistent nausea and vomiting were suspected to be medication-related, leading to volume depletion and prerenal azotemia. Concurrently, the severe anemia raised suspicion for oxidative hemolysis in the setting of known G6PD deficiency, which may have further exacerbated kidney injury through hemoglobinuria-induced tubular stress. Over the course of hospitalization, her renal function gradually improved, with serum creatinine downtrending to 4.95 mg/dL, and her hemoglobin stabilized at 8.3 g/dL by the time of discharge. Both pegloticase and mycophenolate were discontinued, and she was discharged with close outpatient follow-up.

## Discussion

Pegloticase (KRYSTEXXA®) is a pegylated recombinant uricase used for uncontrolled and treatment-refractory gout. By catalyzing the conversion of uric acid to allantoin, uricase therapy generates hydrogen peroxide as a byproduct, creating oxidative stress that can precipitate intravascular hemolysis (and sometimes methemoglobinemia) in patients with G6PD deficiency. Because of this mechanism-based risk, pegloticase is contraindicated in patients with G6PD deficiency, and screening is recommended for patients at risk before treatment is initiated.

Published reports of hemolysis after pegloticase are uncommon, but several cases are documented and share consistent themes: recent pegloticase exposure, underlying or unmasked G6PD deficiency, clinically significant anemia, and, in some cases, concomitant methemoglobinemia [6-9]. Owens et al. (2016) described hemolytic anemia after pegloticase infusion in a patient later found to have G6PD deficiency [6]. Geraldino-Pardilla et al. (2014) reported severe hemolysis with methemoglobinemia after pegloticase in a patient whose initial G6PD testing was falsely negative, highlighting that screening can miss deficiency under certain circumstances, such as during/after hemolysis or transfusion [7]. In contrast, our patient had known G6PD deficiency, yet the diagnosis was still delayed due to normalization of hemolysis markers by the time of presentation. Adashek and Bourji (2018) reported a patient who developed symptomatic anemia approximately one week after pegloticase infusion and was subsequently diagnosed with G6PD deficiency [8]. This case emphasized a delayed presentation and the recognition of this rare but serious adverse effect. Minshar et al. (2019) published a brief case report/letter describing pegloticase-associated hemolysis, adding to the limited post-marketing clinical literature [9]. Across these reports, common features include recent pegloticase exposure, subsequent discovery (or missed detection) of G6PD deficiency, clinically significant anemia, and, in at least one case, concomitant methemoglobinemia.

Our patient developed profound anemia (hemoglobin 5.1 g/dL) and severe AKI (creatinine 9.23 mg/dL) within days of her first pegloticase infusion, a time course that is consistent with prior reports describing presentation within days to approximately one week after exposure. Similar to the 2014 report, our case also illustrates that hemolysis may not present with the “classic” full laboratory pattern by the time of evaluation, particularly when presentation is delayed [8]. A particularly supportive finding in our case was heme-positive urine with no identified red blood cells under the microscope, which indicates hemoglobinuria rather than hematuria. This occurs when free hemoglobin released during intravascular hemolysis exceeds haptoglobin binding capacity and is filtered by glomeruli, providing direct evidence of recent intravascular hemolysis even when serum markers have normalized. This pattern is a well-recognized clue in intravascular hemolysis syndromes and is mechanistically consistent with oxidative hemolysis in G6PD deficiency.

A key interpretive challenge - and the central teaching point of this case - was the absence of overt hemolysis markers (normal haptoglobin and bilirubin) despite severe anemia. This demonstrates that in delayed presentations, normal hemolysis markers do not exclude recent oxidative hemolysis, and clinicians must rely on exposure history, hemoglobinuria, and temporal association. Delayed presentation (four to five days post-infusion) may allow partial biochemical normalization in which bilirubin can be cleared hepatically, and haptoglobin can recover through hepatic synthesis after the acute intravascular hemolytic phase. This pattern is consistent with the concept that uricase-associated oxidative hemolysis may be time-sensitive and that laboratory markers can evolve rapidly [10]. Lactate dehydrogenase (LDH) is nonspecific and can be influenced by systemic inflammation and comorbid conditions (such as rheumatoid arthritis); therefore, mild-to-moderate LDH elevation should be interpreted in the clinical context rather than used as a standalone determinant of hemolysis.

While prior case reports emphasize hemolysis (and sometimes methemoglobinemia), our case highlights the clinically important overlap between hemolysis and kidney injury. Hemoglobinuria from intravascular hemolysis can precipitate acute tubular injury through direct tubular toxicity and cast formation, risks that are magnified in the setting of hypovolemia and reduced renal reserve, particularly relevant in our patient with a solitary kidney and severe vomiting [11]. Severe anemia itself can also lead to ischemic acute tubular necrosis by severely reducing oxygen delivery to the kidneys. This intersection raises the practical point that when profound anemia and AKI co-occur after uricase exposure, clinicians should actively evaluate for hemolysis even if the classic marker pattern is incomplete at presentation.

To our knowledge, only a limited number of cases of pegloticase-associated hemolytic anemia have been reported in the literature, most occurring in patients with underlying or previously unrecognized G6PD deficiency [6-9]. This case highlights two important clinical considerations. First, strict avoidance of pegloticase in patients with G6PD deficiency and adherence to recommended pre-treatment screening are essential to prevent potentially severe oxidative hemolysis [2,3]. Second, in patients presenting several days after exposure, normal haptoglobin and indirect bilirubin levels do not exclude prior hemolysis; clinicians should integrate the exposure timeline and supportive findings such as heme-positive urinalysis with minimal red blood cells into their diagnostic assessment [5].

## Conclusions

Pegloticase can trigger clinically significant oxidative hemolysis in patients with G6PD deficiency, even after a single infusion and with delayed presentation. This case illustrates how hemolysis may be difficult to recognize when patients present several days after exposure, as traditional markers such as haptoglobin and bilirubin may no longer be overtly abnormal. Therefore, a high index of suspicion based on exposure history, medication timing, severe anemia, and hemoglobinuria is essential for accurate diagnosis. Our case also highlights the potential for concurrent complications, including AKI, in the context of severe hemolytic anemia and volume depletion. Ultimately, this report emphasizes the importance of strict pre-treatment G6PD screening and heightened clinical vigilance when evaluating anemia following pegloticase therapy.
